# Inhibitory effect on expression of angiogenic factors by antiangiogenic agents in renal cell carcinoma

**DOI:** 10.1038/sj.bjc.6600152

**Published:** 2002-03-04

**Authors:** H Sasamura, A Takahashi, N Miyao, M Yanase, N Masumori, H Kitamura, N Itoh, T Tsukamoto

**Affiliations:** Department of Urology, Sapporo Medical University School of Medicine, S-1, W-16, Chuo-ku, Sapporo 060-8543, Japan

**Keywords:** inhibition, angiogenic factors, kidney, neoplasm

## Abstract

Since it has been widely recognised that renal cell carcinoma is refractory to standard therapies such as chemotherapy and radiotherapy, a new modality of treatment is needed. One of the potential alternative therapies for renal cell carcinoma may be inhibition of angiogenesis. In this study, we analysed the inhibitory effects of several potential agents on expression of angiogenic factors such as vascular endothelial growth factor and basic fibroblast growth factor, which are the main mediators in angiogenesis of renal cell carcinoma. We used medroxyprogesterone acetate, interferon-alpha, interferon-gamma, minocycline hydrochrolide and genistein, which are known to be antiangiogeneic. Northern blot analyses revealed that, among the five agents examined, genistein had a strong inhibitory effect on expression of vascular endothelial growth factor mRNA and basic fibroblast growth factor mRNA. Medroxyprogesterone acetate and interferon-alpha did not significantly decrease the level of either vascular endothelial growth factor mRNA or basic fibroblast growth factor mRNA. Interferon-gamma and minocycline had mild inhibitory effects on vascular endothelial growth factor mRNA and basic fibroblast growth factor mRNA expression. Genistein also inhibited both vascular endothelial growth factor mRNA and basic fibroblast growth factor mRNA expression after treatment with epidermal growth factor and hypoxia. These findings suggest that one of the mechanisms of the inhibition of angiogenesis by genistein is suppression of the expression of the angiogenic factors vascular endothelial growth factor and basic fibroblast growth factor in renal cell carcinoma.

*British Journal of Cancer* (2002) **86**, 768–773. DOI: 10.1038/sj/bjc/6600152 www.bjcancer.com

© 2002 Cancer Research UK

## 

At present there are no standard effective therapies but surgery for patients with renal cell carcinoma (RCC). RCC is resistant to chemotherapy, radiotherapy and other systemic therapies. Only a minority of patients with RCC can benefit from immunotherapy with interferon (IFN) or interleukin-2. These facts make it frustrating for urologists and oncologists to treat RCC patients. Therefore, a new modality of treatment for patients with RCC is needed.

Angiogenesis is essential for the growth, progression and metastasis of solid tumours (Liota *et al*, 1991; Weidner *et al*, 1991; Folkman, 1996). Most RCCs are characterized by hypervascularity. We have demonstrated that vascular endothelial growth factor (VEGF) and basic fibroblast growth factor (bFGF) play important roles in eliciting angiogenesis in RCC (Takahashi *et al*, 1994). In addition, we have reported that overexpression of VEGF receptors, VEGFR-1 (FLT-1) and VEGFR-2 (KDR), is observed in parallel with that of VEGF in RCC (Takahashi *et al*, 1999). These findings indicate that the VEGF–VEGF receptor pathway is important for angiogenesis in RCC as well as other cancers (Claffey and Robinson, 1996). Therefore, abrogating this pathway can lead to inhibition of tumour growth via suppression of angiogenesis. Indeed, several studies have shown reduction of tumour growth by disrupting this pathway in various tumours. Those disruptions were caused by a neutralizing antibody, receptor antagonist, antisense constructs, or inhibitor of receptor tyrosine-kinases (Kim *et al*, 1993; Claffey *et al*, 1996; Drevs *et al*, 2000).

Several reports have demonstrated that interferon-alpha (IFN-α) and interferon-beta (IFN-β) can down-regulate the expression and production of bFGF, leading to inhibition of angiogenesis in various human cancers (Singh *et al*, 1995; Dinney *et al*, 1998). This observation prompted us to investigate whether there are agents that regulate expression of VEGF as well as bFGF. In this study, we examined the effects of medroxyprogesterone acetate (MPA), IFN-α, interferon-gamma (IFN-γ), minocycline, and genistein. MPA, IFN-α and IFN-γ are clinically administrated against RCC patients and are known to have antiangiogenic effects (Sipos *et al*, 1994; Pepper *et al*, 1996). However, previous studies have shown that these agents produce low response rates in RCC patients. Therefore, we hypothesised that these results would be reasonable if they did not inhibit the expression of VEGF and bFGF in RCC cell lines. We chose these agents to clarify this issue.

Our previous study showed that minocycline inhibits experimental metastasis of mouse renal adenocarcinoma (Masumori *et al*, 1994). In addition, minocycline inhibits angiogenesis *in vivo* and *in vitro* (Tamargo *et al*, 1991; Gilbertson-Beadling *et al*, 1995). These results stimulated us to study whether minocycline could inhibit angiogensis by suppressing VEGF and bFGF expression in RCC. If this drug has such an effect, this could be a novel therapeutic agent.

Genistein, an isoflavone in soybeans, is also antiangiogenic (Fotsis *et al*, 1995). Additionally, genistein inhibits hypoxia-induced VEGF overexpression by suppressing c-*src* activity (Mukhopadhyay *et al*, 1995). However, it remains unclear whether this inhibits angiogenesis by regulating the expression of angiogenic factors in RCC.

Here we report that, among the agents tested, genistein has a strong inhibitory effect on expression of VEGF and bFGF in RCC cell lines *in vitro*.

## MATERIALS AND METHODS

### Cell lines and cultures

Human renal cell carcinoma cell lines SMKT-R-1 and SMKT-R-3, which were established in our laboratory, were maintained in minimal essential medium with D-valine modification medium containing 10% FBS (Miyao *et al*, 1989). Hypoxia was induced by using a BBL GasPak Pouch (Becton Dickinson, Cockeysville, MD, USA), which catalytically reduces O_2_ to an undetectable level.

### Reagents

Epidermal growth factor was purchased from Sigma Chemical Co. (St. Louis, MO, USA). MPA and genistein were from Waco Pure Chemical Industries (Osaka, Japan). Human IFN-α-2b and minocycline were obtained from Schering Plough (Osaka, Japan) and Lederle Ltd. (Tokyo, Japan), respectively. Human IFN-γ was generously provided by Shionogi and Co., Ltd. (Osaka, Japan).

### RNA extraction

Total RNA was obtained from the cell lines using a TRIzol kit (Gibco BRL, Grand Island, NY, USA) according to the procedure recommended by the supplier.

### Northern blot analysis

Twenty micrograms of total RNA was electrophoresed in 1% agarose gels containing formaldehyde and transferred to a NitroPlus membrane (Micron Separation, Inc, Westboro, MA, USA). Hybridisation and washing were performed as described previously (Takahashi *et al*, 1994). Briefly, hybridisation was carried out in 50% formamide, 5× SCC, 5× Denhart's solution, 5 mM EDTA, 0.1% SDS, 10% dextran sulphate and 100 μg ml^−1^ denatured salmon sperm DNA at 42°C for 14–16 h. The filters were washed twice with 0.1× SCC and 0.1% SDS at room temperature for 10 min each and then washed twice at 65°C for 30 min each. All DNA probes were labelled with α-^32^*P*-dCTP (Amersham, Tokyo, Japan) using a random primer labelling system (Boehringer–Mannheim, Mannheim, Germany). After hybridisation, all blots were exposed to Kodak XAR film with an intensifying screen at −80°C. The hybridised probes for VEGF, bFGF and β-actin, were described in a previous report (Takahashi *et al*, 1994). The level of expression of each gene was determined using a Bio-Image-Analyzer (BAS2000; Fujix, Kanagawa, Japan). The percentage of inhibition was determined as follows: % inhibition=[1-(A/B)]×100, where A is nontreated radioactivity (β-actin correction) and B is treated radioactivity (β-actin correction).

## RESULTS

### Effects of various agents on expression of VEGF mRNA and bFGF mRNA in human RCC cell lines

**Figure 1:**
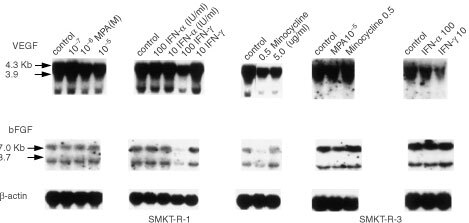
Effects of various agents on the expression of VEGF mRNA and bFGF mRNA in renal cell carcinoma cell lines (SMKT-R-1 and SMKT-R-3). Per cent inhibition was determined by (1−[nontreated radioactivity (β-actin correction)/treated radioactivity (β-actin correction)]×100).

In the first set of experiments, we investigated the effects of MPA, IFN-α, IFN-γ, minocycline and genistein on expression of VEGF mRNA and bFGF mRNA. SMKT-R-1 and SMKT-R-3 cells were incubated in either medium alone or in medium containing various concentrations of agents for appropriate times. Based on previous reports (Tamargo *et al*, 1991; Mukhopadhyay *et al*, 1995; Singh *et al*, 1995; Kim *et al*, 1996) and our preliminary results, we decided the incubation times as follows: genistein for 12 h, MPA and minocycline for 24 h, and IFN-α and IFN-γ for 96 h. These incubation periods as well as concentrations of agents did not affect the cell proliferation (data not shown). We analysed the expression of VEGF mRNA and bFGF mRNA by Northern blot analysis. MPA (10^−5^ M) and IFN-α (100 IU ml^−1^) did not significantly decrease the level of either VEGF mRNA or bFGF mRNA in the cell lines. IFN-γ (100 IU ml^−1^) reduced the expression of VEGF mRNA and bFGF mRNA in SMKT-R-1 cells by 30 and 32%, respectively. In SMKT-R-3 cells, it reduced VEGF mRNA by 45%, but did not alter the expression of bFGF mRNA. Minocycline (0.5 μg ml^−1^) also reduced VEGF mRNA and bFGF mRNA in SMKT-R-1 cells by 45 and 22%, respectively. It did not reduce either mRNA level in SMKT-R-3 cells (Figure 1A

and Table 1

**Table 1:**
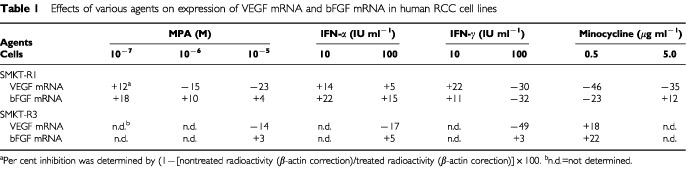
Effects of various agents on expression of VEGF mRNA and bFGF mRNA in human RCC cell lines

). Genistein significantly inhibited the expression of VEGF mRNA in a dose-dependent manner. A high concentration (100 μg ml^−1^) of genistein inhibited the expression of VEGF mRNA by 55% in SMKT-R-1 and 30% in SMKT-R-3. Genistein also induced 30–50% decreases in the level of bFGF mRNA in both cell lines (Figure 2A,B

**Figure 2:**
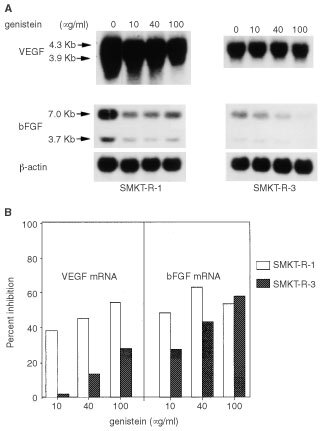
(**A**,**B**) The effect of genistein on the expression of VEGF mRNA and bFGF mRNA in SMKT-R-1 and SMKT-R-3 cell lines. Both cell lines were incubated for 12 h with serum-free medium containing 4, 40, 100 μg ml^−1^ of genistein.

).

### Effect of genistein on expression of VEGF mRNA and bFGF mRNA in human RCC cell lines treated with EGF and hypoxia

**Figure 3:**
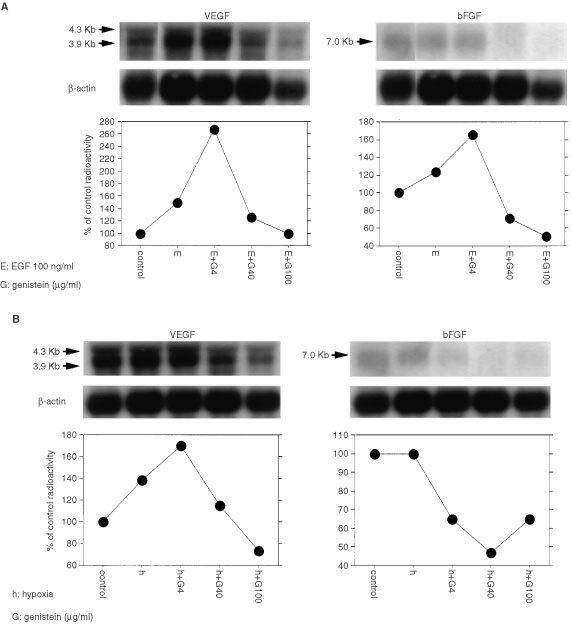
(**A**, **B**) The effect of genistein on the expression of VEGF mRNA and bFGF mRNA in SMKT-R-3 cells treated with EGF or hypoxia. These cells were incubated with serum-free medium and with 0, 4, 40, 100 μg ml^−1^ of genistein with EGF (100 ng ml^−1^) or under hypoxic conditions for 12 h.

Since genistein had a stronger inhibitory effect on expression of VEGF mRNA and bFGF mRNA, we performed further experiments using genistein in SMKT-R-3 cells. Several experiments have demonstrated that expression of VEGF and bFGF is upregulated by various factors, including cytokines and hypoxia (Tsai *et al*, 1995; White *et al*, 1995; Claffey and Robinson, 1996). Therefore, we investigated whether EGF and hypoxia could induce VEGF and bFGF expression in human RCC cell lines and whether genistein could regulate the expression of these angiogenic factors under stimulation of EGF and hypoxia. SMKT-R-3 cells were treated with various concentrations of genistein (4, 40, 100 μg ml^−1^) in the presence of EGF (100 ng ml^−1^) for 12 h. Under these conditions, EGF increased the levels of VEGF mRNA and bFGF mRNA by about only 1.4- and 1.2-fold respectively, compared with no treatment (control). Genistein inhibited EGF-treated VEGF mRNA and bFGF mRNA expression in a dose-dependent manner (Figure 3A

). SMKT-R-3 cells were cultured with various concentrations of genistein (4, 40, 100 μg ml^−1^) under hypoxic conditions for 12 h. VEGF mRNA exhibited only a 1.4-fold increase in response to hypoxia in this cell line. bFGF mRNA was not induced by hypoxia. Expression of both VEGF mRNA and bFGF mRNA was down-regulated by genistein (Figure 3B).

## DISCUSSION

Angiogenesis occurs as a result of complex multi-step processes such as activation of endothelium, destruction of basement membrane by proteolytic enzymes, migration and proliferation of endothelial cells, and formation of tubular structures (Folkman and Shing, 1992). These steps are stimulated by the interactions between tumour cells releasing angiogenic factors and their receptors. Tumour angiogenesis can be inhibited by blocking more than one of these steps. Prevention of tumour angiogenesis involves inhibition of synthesis of angiogenic factors and blocking of their receptors (Barillie *et al*, 1995; O'Brien and Harris, 1995). Until now there have been few reports regarding the inhibition of expression and production of angiogenic factors (Sidky and Borden, 1987; Singh *et al*, 1995). Here we examined whether certain agents could suppress the expression of VEGF mRNA and bFGF mRNA, which are the main angiogenic factors in RCC (Takahashi *et al*, 1994).

Among the agents tested, genistein showed more prominent suppression of the expression of VEGF mRNA and bFGF mRNA. Genistein, an isoflavone in soybeans, was originally recognised as a weak oestrogen and also an oestrogen antagonist at higher concentrations (Zava and Deuw, 1994). Genistein inhibits the tumour growth in various cancers *in vitro* and *in vivo* (Fotsis *et al*, 1995). In addition, it is an inhibitor of angiogenesis (Fotsis *et al*, 1995). Although the mechanism responsible for the inhibition described above remains unclear, accumulating studies have shown that genistein has multiple antitumour functions. Those include inhibition of tyrosine kinase activity (Akiyama *et al*, 1987), topoisomerase II (Okura *et al*, 1988) and steroid metabolizing enzymes such as 17β-hydroxysteroid oxidoreductase (Mäkelä *et al*, 1998), P-450 aromatase (Adlercreutz *et al*, 1993) and 5-α reductase (Evans *et al*, 1995), induction of differentiation (Record *et al*, 1997), G2/M arrest, induction of p21^WAF1/CIP1^ and apoptosis (Shao *et al*, 1998). With regard to angiogenesis, genistein blocks the expression of VEGF induced by hypoxia by inhibiting src tyrosine kinase in some cancer cell lines (Mukhopadhyay *et al*, 1995). Thus, we were interested in whether genistein could suppress the expression of VEGF and bFGF, which are the main angiogenic factors in RCC. In our study, genistein down-regulated the expression of both genes under all conditions examined, including EGF and hypoxic treatments. Recently Shao *et al* (1998) reported that genistein inhibited angiogenesis by decreasing vessel density and decreasing the level of VEGF as well as transforming growth factor-β1 in a human breast cancer cell. Regarding bFGF, to our knowledge this is the first study to demonstrate that genistein also has a strong inhibitory effect on expression of bFGF mRNA in RCC. This finding gives us important information about treatment for RCC, because a recent report showed a significant role of bFGF in regard to development of metastasis (Slaton *et al*, 2001). Therefore, inhibition of bFGF as well as VEGF by genistein may lead to prevention of metastasis.

MPA is known to have an antiangiogenic effect via suppression of plasminogen activator activity (Blei *et al*, 1993). In our study, MPA did not induce a significant decrease in the level of either VEGF mRNA or bFGF mRNA in the concentrations employed. This finding is consistent with the report of Kim *et al* (1996) showing that VEGF mRNA expression is not altered by MPA or oestradiol in an *in vivo* model of endometrial carcinoma.

Our previous study showed that minocycline inhibits *in vitro* invasion and experimental metastasis of mouse renal adenocarcinoma by inhibiting type IV collagen degradation (Masumori *et al*, 1994). However, the mechanism of action remains unclear. Thus, we investigated the effect of minocycline on the expression of VEGF mRNA and bFGF mRNA. Minocycline (0.5 μg ml^−1^) showed a moderate inhibitory effect on VEGF mRNA and bFGF mRNA expression only in SMKT-R-1 cells. This result indicated that this phenomenon might possibly be specific for SMKT-R-1 cells.

IFNs are multifunctional cytokines that regulate immune responses as well as antiviral and antitumour activities. IFNs are also inhibitors of angiogenesis. IFN-α and IFN-β have been shown to have anti-angiogenic activity by suppressing the expression and production of bFGF in various human tumour cells, including RCC (Singh *et al*, 1995; Dinney *et al*, 1998). These findings prompted us to examine whether INFs could down-regulate the expression of VEGF as well as bFGF. However, in our study, neither the expression of VEGF mRNA nor that of bFGF mRNA was altered by IFN-α, even though we did experiments under the same conditions (time for treatment and drug concentration) as previous researchers did. The finding that IFN-α failed to reduce expression of VEGF mRNA is consistent with that of a previous report (Dinney *et al*, 1998). However, regarding bFGF, our result was in disagreement with those of other reports (Singh *et al*, 1995; Dinney *et al*, 1998). One explanation may be the difference of subunits of IFN-α, because we used IFN-α-2b instead of the IFN-α-2a used in other experiments. Another reason could be the difference of cell lines examined.

Several experiments have demonstrated that expression of VEGF and bFGF is up-regulated by various factors, including cytokines such as EGF, TGF-α, platelet-derived growth factors, and hypoxia (Claffey and Robinson, 1996). Hypoxia, in particular, is a strong inducer of up-regulation of the VEGF gene. It induces 1.1- to 28.9-fold VEGF mRNA expression in normal and tumour cells *in vitro* (White *et al*, 1995; Claffey and Robinson, 1996). Hypoxia-stimulated VEGF expression is due to increases in both transcriptional activity and mRNA stabilisation (Ikeda *et al*, 1995; Levy *et al*, 1995, 1996). In our study, hypoxia did not induce significant up-regulation of VEGF mRNA in the cell lines examined. This may have been due to differences of sensitivity to hypoxia. Some reports have demonstrated that human tumour cells with high expression of VEGF mRNA exhibit persistent mRNA stabilisation through oncogenic activation of tyrosine kinase and Ras protein, and fail to further stabilise VEGF mRNA in response to hypoxia (White *et al*, 1995, 1997). Furthermore, they observed that the higher the basal abundance of the VEGF mRNA that tumour cell lines exhibited, the less responsive to hypoxia they were. Since the cell lines that we used, SMKT-R-1 and R-3, express VEGF mRNA at a level higher than the glioblastoma multiforma cell line U-251MG, which is known to contain high levels of VEGF mRNA (Takahashi *et al*, 1994), our results may be consistent with those findings.

Considering the effect of genistein on growth inhibition in RCC cell lines (unpublished data), it may be a novel therapeutic agent for RCC patients. However, since inhibition of the expression of VEGF and bFGF by genistein was incomplete, genistein alone may be insufficient for a large metastatic RCC. Therefore, genistein may be effective for chemoprevention for patients who are at high risk for RCC (i.e. von Hippel-Lindau disease patients), or prevention of metastasis for post-surgery patients.
